# Lateral Heterometal Junction Rectifier Fabricated by Sequential Transmetallation of Coordination Nanosheet**

**DOI:** 10.1002/anie.202318181

**Published:** 2024-01-23

**Authors:** Choon Meng Tan, Naoya Fukui, Kenji Takada, Hiroaki Maeda, Ekaterina Selezneva, Cédric Bourgès, Hiroyasu Masunaga, Sono Sasaki, Kazuhito Tsukagoshi, Takao Mori, Henning Sirringhaus, Hiroshi Nishihara

**Affiliations:** ^1^ Research Institute for Science and Technology Tokyo University of Science 2641 Yamazaki Noda Chiba 278 8510 Japan; ^2^ WPI International Center for Materials Nanoarchitectonics (WPI-MANA) National Institute for Materials Science (NIMS) Namiki 1-1 Tsukuba 305-0044 Japan; ^3^ Cavendish Laboratory University of Cambridge JJ Thomson Avenue Cambridge CB3 0HE UK; ^4^ International Center for Young Scientists (ICYS) National Institute for Materials Science (NIMS) Namiki, Tsukuba 305-0044 Japan; ^5^ Japan Synchrotron Radiation Research Institute (JASRI) 1-1-1 Kouto, Sayo-cho Sayo-gun Hyogo 679-5198 (Japan); ^6^ Faculty of Fiber Science and Engineering Kyoto Institute of Technology 1 Matsugasaki Hashikami-cho Sakyo-ku Kyoto 606-8585 Japan; ^7^ RIKEN SPring-8 Center 1-1-1 Kouto Sayo-cho Sayo-gun Hyogo 679-5148 Japan

**Keywords:** Coordination Nanosheet, Lateral Heterojunction, Nanostructures, Rectifier, Transmetallation

## Abstract

Heterostructures of two‐dimensional materials realise novel and enhanced physical phenomena, making them attractive research targets. Compared to inorganic materials, coordination nanosheets have virtually infinite combinations, leading to tunability of physical properties and are promising candidates for heterostructure fabrication. Although stacking of coordination materials into vertical heterostructures is widely reported, reports of lateral coordination material heterostructures are few. Here we show the successful fabrication of a seamless lateral heterojunction showing diode behaviour, by sequential and spatially limited immersion of a new metalladithiolene coordination nanosheet, Zn_3_BHT, into aqueous Cu(II) and Fe(II) solutions. Upon immersion, the Zn centres in insulating Zn_3_BHT are replaced by Cu or Fe ions, resulting in conductivity. The transmetallation is spatially confined, occurring only within the immersed area. We anticipate that our results will be a starting point towards exploring transmetallation of various two‐dimensional materials to produce lateral heterojunctions, by providing a new and facile synthetic route.

## Graphical abstract

A lateral heterojunction, fabricated by spatially confined transmetallation of a precursor zinc(II) benzenehexathiolato coordination nanosheet with Fe(II) and Cu(II) ions is reported. Structural differences between the transmetallated and the as‐prepared nanosheets lead to physical and chemical differences. Band structure bending across the interface of the transmetallated nanosheets results in the observed rectifying behavior.

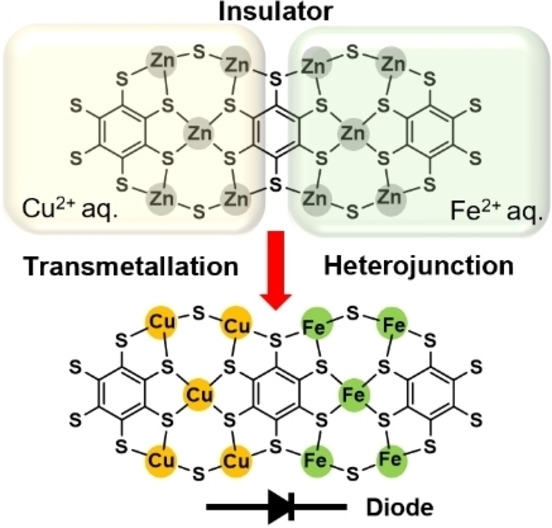

Electronically conducting two‐dimensional (2D) materials such as graphene^[1]^ and transition metal dichalcogenides^[2]^ (TMDCs) are fascinating research targets in both physics and chemistry as their unique topological properties and functionalities open new scientific and technological fields. The combination of different 2D materials expands the variation of electro‐, photo‐, and magneto‐functions, realising a variety of device applications inaccessible by single material systems.^[3]^ Heterostructures of 2D materials can be fabricated in two ways: as vertical heterojunctions or lateral heterojunctions.^[4]^ Confinement of charge carriers within the two‐dimensional plane enhances physical properties: hence unique in‐plane devices such as p‐n diode,^[5]^ Schottky diode,^[6]^ photocurrent generation,^[7]^ electroluminescence^[8]^ and CMOS inverter^[9]^ can be realised. However, construction of the lateral heterojunction is challenging compared with the vertical heterojunction, which can be simply achieved by stacking two flakes of different materials with the naturally obtained atomically flat surfaces.^[10]^

Nowadays, conductive 2D materials are explored not only in inorganic materials but also in organic materials, which are in most cases light and flexible.[^11^, ^12^] Coordination nanosheets, composed of planar organic ligands coordinating to metal ions with a square planar geometry, have been a representative conductive 2D organic material since our report of the synthesis of nickel benzenehexathiol (BHT) nanosheet Ni_1.5_BHT in 2013.^[13]^ The combination of BHT and various transition metals can afford M/BHT family of nanosheets, porous M_1.5_BHT[^14^, ^15^, ^16^] and non‐porous M_3_BHT[^17^, ^18^, ^19^, ^20^, ^21^] with wide‐ranging physical and chemical properties such as superconductivity,^[20]^ topological spin glass^[18]^ and redox control of conductivity.^[22]^ They have also been incorporated into devices as hole buffer layer,^[23]^ photodetectors^[24]^ or pseudocapacitors.^[19]^

Facile postsynthetic modification is an advantage of coordination nanosheets. Metal centres in coordination materials can be replaced with other metal ions, in a postsynthetic transmetallation reaction by only immersing in the corresponding metal salt solution.^[25]^ This method allows the synthesis of materials that cannot be synthesized by direct reaction: such as when undesirable metal‐ligand side reactions occur, kinetic coordination competition within metal ion mixtures. The previous example is Schlüter and co‐workers’ work which reported that bis(terpyridine)zinc nanosheet was transmetallated with Co(II), Pb(II) and Fe(II) to give the respective bis(terpyridine)metal nanosheets.^[26]^ Since bis(terpyridine)metal nanosheet generally possesses poor electrical conductivity, conductive nanosheets obtained by transmetallation are required for future application to electronics. For example, BHT‐based coordination nanosheets may affords various conducting nanosheets after transmetallation and moreover lead to heterojunction by spatially controlled immersion into different metal solutions. However, research of transmetallation as a method to fabricate heterostructures is still in an early stage.

In this study, we attempted to synthesize lateral heterojunction of conducting metalladithiolene nanosheets utilising sequential transmetallation. For this purpose, we first synthesized and characterized Zn‐BHT coordination nanosheet. We found that Zn forms nonporous Zn_3_BHT nanosheet. Next, we investigated the transmetallation of Zn_3_BHT sheet with Cu(II) and Fe(II). Both metals replaced Zn to give transmetallated nanosheets tmCu and tmFe, respectively. Finally, we synthesized lateral tmFe/tmCu heterojunction by sequential transmetallation of Zn_3_BHT. A seamless boundary was realised with physical and electrical connection maintained, taking advantage of transmetallation method. We found that this heterojunction shows a rectifying behaviour. Thermoelectric measurements and Kelvin force microscopy reveal that a p‐p junction caused the rectification.

Initial trials of the liquid‐liquid interfacial synthesis of Zn/BHT nanosheet, by layering a solution of Zn(OAc)_2_ on top of a dichloromethane solution of BHT at room temperature and stoichiometric amount of (Zn^2+^: BHT=3 : 1) were done. Allowing the reaction to proceed for 24 hours under inert atmosphere gave a thick opaque white film. We measured the powder X‐ray diffraction (PXRD) pattern of the film as shown in Figure 1b. Broad diffraction peaks were observed which show that periodicity occurs only in small domains. To obtain higher crystalline Zn/BHT suitable for structure determination, Zn/BHT was synthesized at higher temperature (45 °C), replacing dichloromethane with chloroform (Figure 1a). As shown in Figure 1b, the crystallinity of the resulting Zn/BHT was greatly improved. This improvement of the crystallinity at higher temperature is commonly seen in the synthesis of M_3_BHT nanosheets such as Fe_3_BHT and Cu_3_BHT.[^17^, ^20^] It can be explained by the thermally enhanced reversibility of complexation, which promotes the formation of thermodynamically stable crystal structure. The diffraction peaks of Zn/BHT provided enough information to determine its structure as depicted in Figure 1c. The pattern was reproduced by an AB slipped parallel stacking of nonporous structure, Zn_3_BHT, where one monolayer was simulated as a flat layer, and the next layer translated in a slipped parallel fashion. This gave a Zn_3_BHT structure with unit cell parameters *a*=*b*=8.7 Å, *c*=7.1 Å, α=β=90°, γ=120° with a translation of the B‐layer 0.8 units in the *a*‐direction and 0.45 units in the *b*‐direction (Figure 1c). The in‐plane structure of Zn_3_BHT is similar to that of other M_3_BHT (M=Mn, Fe, Ni, Cu) but different in that the other M_3_BHT materials adopt the AA stacking mode.[^17^, ^18^, ^19^, ^20^, ^21^] The crystallinity of Zn_3_BHT was affected by the type of anion in the salt. Zn(OAc)_2_, Zn(BF_4_)_2_, and Zn(NO_3_)_2_ afforded highly crystalline Zn_3_BHT while ZnCl_2_ afforded poorly crystalline Zn_3_BHT (Figure S1). It is also noteworthy that a zinc ion in Zn_3_BHT takes square‐planar geometry while zinc ions generally prefer tetrahedral geometry.

**Figure 1:**
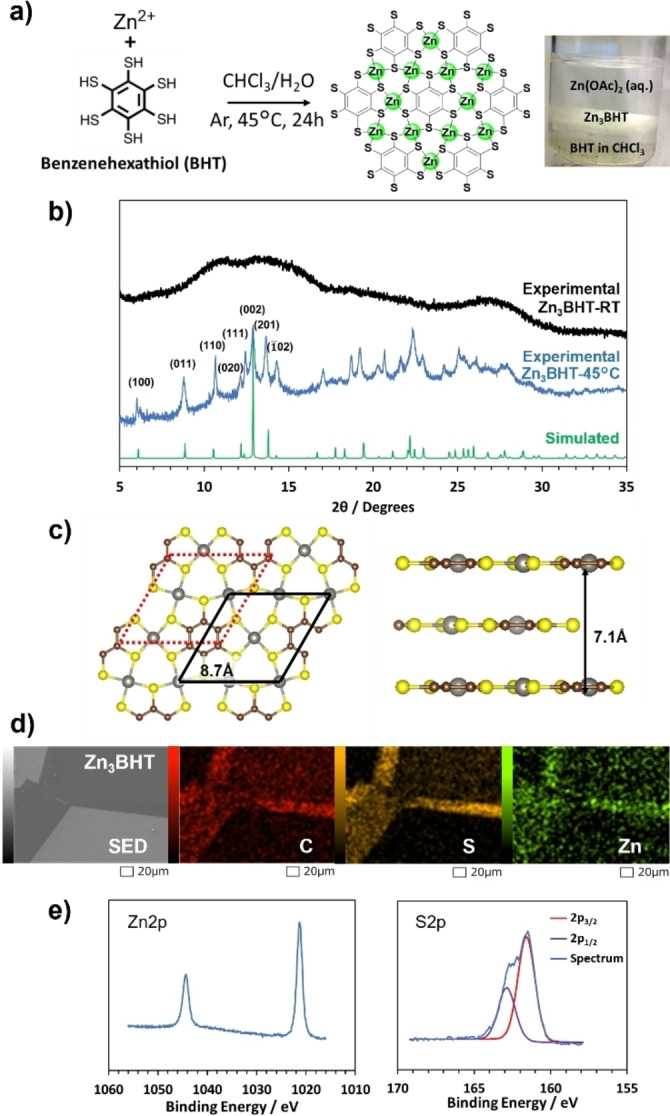
Characterization of Zn_3_BHT. a, Schematic and photo of Zn_3_BHT synthesis. b, Experimental and simulated PXRD patterns of Zn_3_BHT (λ=0.8 Å), synthesized at room temperature and 45 °C. c, model structures of Zn_3_BHT viewed along *c*‐ (left), and *a*‐axes (right). The unit cell is drawn with black solid lines. The red dot lines show the corresponding unit cell of the slipped neighboring layer. d, SEM/EDS elemental maps of Zn_3_BHT nanosheet. e, Narrow X‐ray photoelectron spectra of Zn_3_BHT.

SEM images (Figure 1d) show the monolithic structure of Zn_3_BHT and the EDS elemental maps confirm the uniform distribution of Zn, S and C in the sheet structure. These images confirm that the nanosheets are composed of Zn(II) ions and BHT ligands coordinated to form Zn_3_BHT at the confined liquid‐liquid interfacial region resulting in a flat topography with 30 nm thickness as shown in AFM images (Figure S2).

X‐ray photoelectron spectroscopy (XPS) of Zn_3_BHT indicates the presence of each element Zn and S (Figure 1e). The S2p narrow spectrum can be deconvoluted into a pair of peaks which are assigned to 2p_3/2_ at 161.6 eV and 2p_1/2_ at 162.9 eV. The atomic ratio of Zn : S is 1 : 1.6, which is close to the ratio 1 : 2 implied by the nonporous structure, Zn_3_BHT.

Metal exchange reactions of Zn_3_BHT were conducted using Fe(II) and Cu(II) ions. Samples of Zn_3_BHT immobilised on SiO_2_/Si substrate were immersed in 50 mM salt solution for 3 days (Figure 2a). The residual metal salts were thoroughly washed with a 1 : 1 solution of water:ethanol. The Zn_3_BHT after transmetallation by each metal ion solution (tmM) was investigated with SEM/EDS, XRD, Raman spectroscopy, and electrical measurements. The element maps of the tmM nanosheets (M=Cu, Fe) are shown in Figures 2b and 2c. For this EDS measurement, the Zn_3_BHT was partially immersed for the clear comparison between pristine and immersed Zn_3_BHT samples (Figure 2a). Cu and Fe were uniformly distributed in the immersed region of tmCu and tmFe, respectively, while they are absent in the pristine region. On the other hand, according to the EDS analyses, Zn was not detected in the immersed region (Figures S3 and S4). Carbon and sulphur are uniformly distributed over both pristine and immersed regions. This observation suggests the successful transmetallation of Zn_3_BHT into corresponding tmM by exchanging zinc ions and iron or copper ions with the BHT framework maintained.

**Figure 2:**
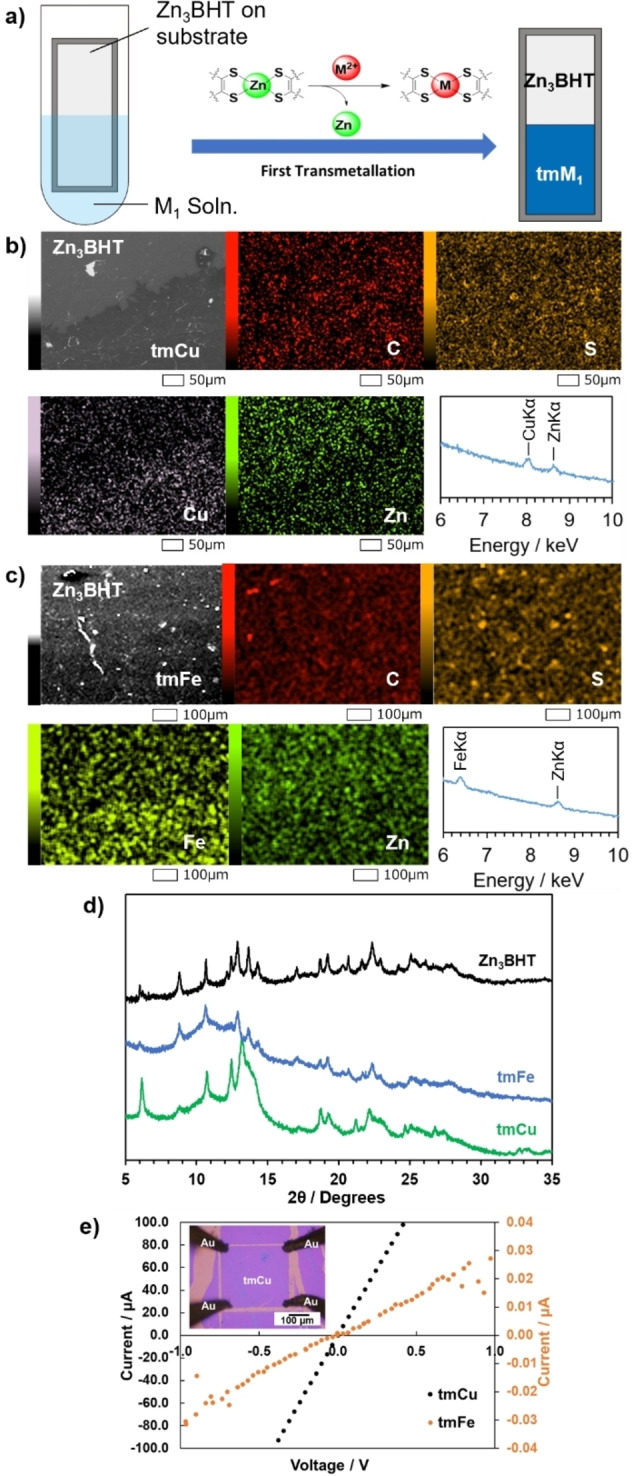
Characterization of tmM. a, Schematic illustration of the synthesis of tmM/Zn_3_BHT heterojunction. b, c, SEM/EDS elemental maps of (b) tmCu, and (c) tmFe nanosheets, d, PXRD patterns of tmM together with Zn_3_BHT before transmetallation (λ=0.8 Å). e, *I*–*V* curves of tmM nanosheets measured by four‐point probe method.

The atomic structures of tmCu and tmFe after the transmetallation were obtained by studying their PXRD patterns (Figure 2d). The position of diffraction peaks of tmFe is the same as that of Zn_3_BHT, implying that they share the same unit cell. On the other hand, the intensity of the peaks differs between tmFe and Zn_3_BHT, with broader peaks in tmFe than in Zn_3_BHT. These results suggest that tmFe maintains the framework originating from Zn_3_BHT (with Fe ions replacing Zn ions) and that during the transmetallation slight disorder was introduced in the sheet. Notably, the structure of tmFe (AB stacking) is hence different from that of previously reported Fe_3_BHT (AA stacking).^[17]^ The situation is different in tmCu, as the resulting structure of the material resembles that of Cu_3_BHT, with the (001) peak shifting from a 2θ value of 12.9° in Zn_3_BHT to 13.2° in tmCu, which is different from 13.5° in Cu_3_BHT directly synthesized from Cu(II) and BHT.[^20^, ^21^, ^27^] This implies that while the in‐plane structure of tmCu is similar to that of Cu_3_BHT, the interlayer distance of tmCu (3.49 Å) is in‐between that of Zn_3_BHT (3.58 Å) and Cu_3_BHT (3.43 Å). A possible explanation is that the structure of tmCu is Cu‐substituted Zn_3_BHT with AB slipped stacking and an expanded interlayer distance, a new phase of Cu/BHT group.

Four‐point‐probe electrical conductivity measurement was performed on Zn_3_BHT and tmM nanosheets. Although the M_3_BHT series reported so far are well‐known to be conductive coordination nanosheets,[^17^, ^18^, ^19^, ^20^] Zn_3_BHT is an insulator as shown in Figure S5. This can be attributed to the closed‐shell electronic structure of the Zn(II) ion, from which it is difficult to generate free electrons or holes. On the contrary, once Zn ions are exchanged with other transition metal ions, the tmM nanosheets become electrical conductors. Their electrical conductivity ranges from 3.06 ×10^−3^ S cm^−1^ for tmFe to 26.6 S cm^−1^ for tmCu (Figure 2e). We also investigated the thermoelectric behaviour of tmM. tmCu is p‐type with a Seebeck coefficient of about +15 μV K^−1^ at room temperature. This value is 2–3 times as large as that of Cu_3_BHT previously reported.[^27^, ^28^] The reduced conductivity and enhanced Seebeck coefficient could potentially reflect differences in the electronic structure between tmCu and Cu_3_BHT due to the slipped AB vs AA stacking, but they may also be manifestations of a higher concentration of defects in the films prepared by transmetallation, such as defects induced by residual Zn. tmFe is also p‐type with Seebeck coefficient of about +100 μV K^−1^ at room temperature, which is a typical magnitude for a semiconductor (Figure S6). The larger Seebeck coefficient implies its lower carrier concentration compared to tmCu.

tmM nanosheets possess a variety of electrical characteristics as mentioned above. This inspired us to combine the two types of tmM to fabricate electronic devices by immersing Zn_3_BHT sequentially in two different metal ion solutions. Thin Zn_3_BHT nanosheet immobilised on SiO_2_/Si substrate was partially immersed in 50 mM Cu(II) or 50 mM Fe(II) solution for 3 days. A boundary between Zn_3_BHT and tmCu or tmFe could be observed optically (Figure S7), suggesting that transmetallation proceeds exclusively at the immersed region which enables its facile spatial control. The non‐immersed area containing pristine Zn_3_BHT was subsequently immersed in 50 mM Fe(II) solution for 3 days (Figure 3a). Scanning electron microscopy was performed to image the boundary area of a tmFe/tmCu lateral heterojunction formed using the sequential‐transmetallation method (Figure 3b). The tmFe/tmCu boundary showed clear contrast in the secondary electron image, where tmCu was dark and tmFe was bright, and matches the elemental distribution of the respective elements. The SEM‐EDS mapping images and energy spectrum near the boundary show that Zn is absent all around the junction (Figure S8). Cu was distributed over the bottom half of the field of view while Fe was found over the upper half of the EDS image where Cu is absent. Therefore, we conclude that a lateral heterojunction of tmCu and tmFe was successfully formed without destroying the sheetlike morphology of the original Zn_3_BHT.

**Figure 3:**
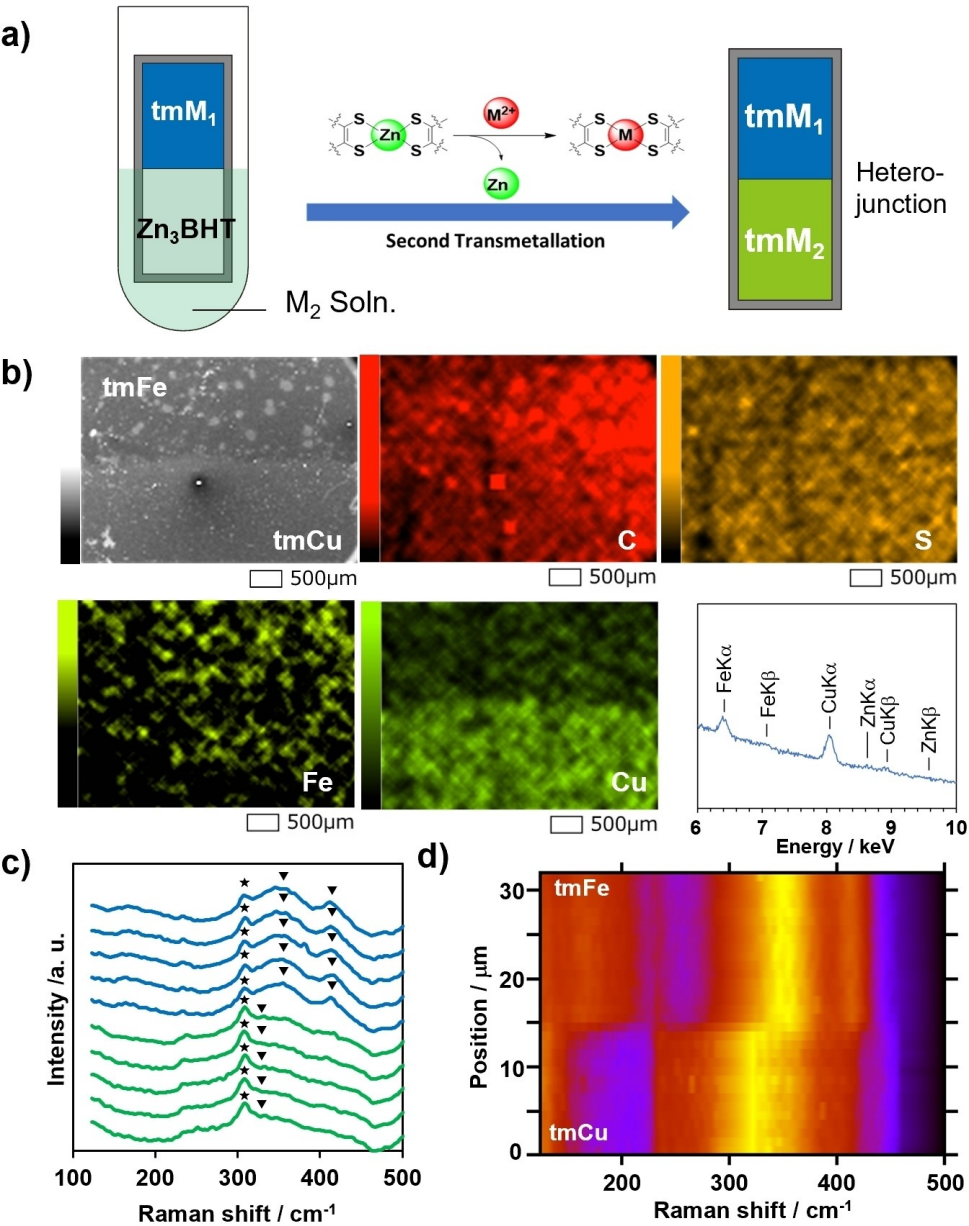
Fabrication of tmM lateral heterojunction. a, Schematic of the fabrication of heterometal junction tmFe/tmCu. b, SEM image, EDS maps of tmFe/tmCu heterojunction, and EDS spectrum of the mapped area. The full spectrum is shown in Figure S8. c, Spatially resolved Raman spectra taken every 1 μm across tmFe/tmCu heterojunction from tmCu region (green lines, bottom) to tmFe region (blue lines, top). The triangles show remarkable peaks and the stars shows the peak from the silicon substrate. d, 2D plot of Raman intensity against Raman shift. The intensity was normalized to the peak top of each region. The silicon peak at 305 cm^−1^ was analytically removed for the clarity by interpolation.

Raman spectra across the tmFe/tmCu heterojunction (Figure 3c) revealed the formation of a seamless junction. A broad peak at 320 cm^−1^ was found in tmCu region while sharp peaks at 356 cm^−1^ and 409 cm^−1^ was found in tmFe region. These peaks are likely to originate from corresponding M−S vibration compared with previous studies.^[27]^ Figure 3d shows the Raman intensity taken at intervals of 1 μm across the tmFe/tmCu junction visualised as 2D colour plot. The boundary around the position of 15 μm is estimated to be ≤1 μm.

The *I*–*V* curves of the lateral heterojunctions were measured at room temperature under Ar atmosphere by the 2‐probe method, using Au tips (Figure 4a). The boundary area in the middle can be roughly visualised with the slight contrast in the images after transmetallation. Here we describe as *V*_ij_ the voltage of tip i with respect to tip j. *I*_ij_ is the current flowing from tip i to tip j, likewise. From Figure 4a, the *I*–*V* curve of *I*_21_ against *V*_21_, measurement across the junction, reveals non‐linear rectifying behaviour across the junction. Current abruptly increased beyond +0.4 V while linear *I*–*V* characteristic was found in the backward direction. Confirmation that this behaviour is not due to Schottky barriers between the Au tips and the respective tmM nanosheets is given by almost ohmic *I*–*V* curves measured between the 2 probes within each respective tmFe or tmCu region (Figure S9). Therefore, we can conclude that it is the tmFe/tmCu junction that induces the rectifying behaviour.

**Figure 4:**
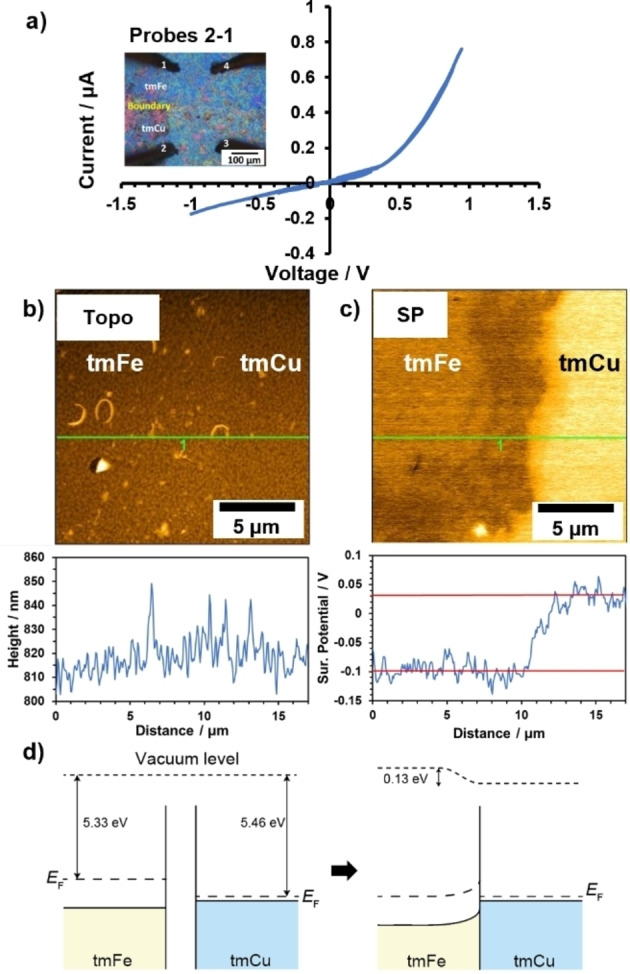
Electrical characterization of tmFe/tmCu heterojunction. a, Rectifying I–V curves of tmFe/tmCu heterojunction. b, AFM topography image and c, KFM surface potential mapping image of tmFe/tmCu on Au/mica substrate. d, Band diagram of tmFe and tmCu before (left) and after (right) the formation of the junction. The conduction bands are omitted for clarity.

To further investigate the electrical properties of both tmFe and tmCu, Kelvin force microscopy (KFM) was performed on the nanosheets, obtaining the surface potential. Figure 4b shows the topographic image of the tmFe/tmCu junction measured by AFM. There was no significant height difference across the boundary between tmCu and tmFe even though clear colour contrast was observed in the optical microscope image of the scanned region (Figure S10). Hence, the transmetallation process in different solutions did not change the morphology of the film. Figure 4c shows the surface potential (SP) map of the same area as Figure 4b, measured simultaneously by KFM. An obvious clear boundary was observed on the SP map, in contrast to the topographical image. Given that the work function of Au surface is 5.30 eV,^[29]^ the work function of tmFe and tmCu was calculated to be 5.33 eV and 5.46 eV respectively, judging from SP profile across tmM/Au boundary (Figure S11). The SP profile shows that the SP changes within 2 μm of the boundary.

These results of electrical conduction and KFM measurements are well explained by considering the formation of p‐p junction at the tmFe/tmCu lateral junction. The band diagrams before and after junction are depicted in Figure 4d. Since both tmCu and tmFe are p‐type semiconductors, their Fermi levels are located above their valence bands. The hole concentration of tmCu is expected to be much larger than tmFe judging from the smaller Seebeck coefficient and the larger conductivity of tmCu. Therefore, the Fermi level of tmCu will be almost on the top of the valence band, whereas that of tmFe will be located away from the top of the valence band (Figure 4d). The difference in their work function leads to the mismatch of the Fermi level by 0.13 eV. The different Fermi level is compensated by the electron transfer from tmFe to tmCu which causes a depletion layer in the tmFe side of the junction. Consequently, a built‐in potential of 0.13 eV is generated and the heterojunction works as a rectifier. The experimental results suggested that a positive forward bias of 0.4 V applied to tmCu with respect to tmFe is required for the current to start flowing, which can be attributed to the consumed voltage drop between the junction and the probes.

In conclusion, we have successfully fabricated a seamless heterojunction (within 1 μm) showing diode behaviour, by sequential and spatially limited immersion of a new coordination nanosheet Zn_3_BHT into aqueous Cu(II) and Fe(II) ion solutions. Insulating Zn_3_BHT undergoes transmetallation while maintaining its BHT framework, with the sequential transmetallation process resulting in conducting tmFe/tmCu heterojunctions. tmFe exhibits p‐type conduction with Seebeck coefficient of +100 μV K^−1^ and work function of 5.33 eV, while tmCu exhibits p‐type conduction with Seebeck coefficient of +15 μV K^−1^ and work function of 5.46 eV respectively.

These results highlight the potential of Zn_3_BHT, whose versatility is showcased by how its characteristics can be changed easily without special equipment. Zn_3_BHT enables, for example, an integrated circuit all made from a single coordination nanosheet and drawn by inkjet printing method without patchworking different kinds of coordination nanosheets and other materials. Therefore, our findings show that transmetallation of two‐dimensional materials can be a powerful new avenue towards fabricating lateral heterostructures while avoiding labour‐intensive processes.

## Supporting Information

Including experimental details, materials, X‐ray diffraction data, Raman spectra, TEM and SEM images, calculation details.

## Conflict of interest

The authors declare no conflict of interest.

## Supporting information

As a service to our authors and readers, this journal provides supporting information supplied by the authors. Such materials are peer reviewed and may be re‐organized for online delivery, but are not copy‐edited or typeset. Technical support issues arising from supporting information (other than missing files) should be addressed to the authors.

Supporting Information

## Data Availability

The data that support the findings of this study are available from the corresponding author upon reasonable request.
