# Supplementary material for: Lateral Heterometal Junction Rectifier Fabricated by Sequential Transmetallation of Coordination Nanosheet
Source: Angew Chem Int Ed Engl. 2024 Jan 23;63(9):e202318181. doi: 10.1002/anie.202318181 (PMC11497271; doi:10.1002/anie.202318181)
Supplement: Supplementary file 1 — Supporting Information [file ANIE-63-e202318181-s001.pdf]

Angewandte Chemie
Eine Zeitschrift der Gesellschaft Deutscher Chemiker

Supporting Information

Lateral Heterometal Junction Rectifier Fabricated by Sequential Transmetallation of Coordination Nanosheet

*C. M. Tan, N. Fukui, K. Takada, H. Maeda, E. Selezneva, C. Bourgès, H. Masunaga, S. Sasaki, K. Tsukagoshi, T. Mori, H. Sirringhaus, H. Nishihara**

Table of Contents

I.	General Experimental Details	S2
II.	Experimental Procedures	S3
III.	Experimental Results	S5
IV.	Reference	S13

I. General Experimental Details

Materials

$Zn(OAc)_2 \cdot 2H_2O$, $Cu(NO_3)_2 \cdot 3H_2O$, $FeSO_4 \cdot 7H_2O$, dichloromethane, chloroform, and ethanol were purchased from Kanto Chemical Co., Inc. Water was purified using the Milli-Q purification system (Merck KGaA). All solvents were degassed by purging with nitrogen before use. Benzenehexathiol (BHT) was synthesized according to the literature[S1]. Silicon (100) wafers (with 285 nm thermally grown oxide) were purchased from Furuuchi Chemicals. Au/mica substrates were purchased from Geomatec Inc.

Apparatus

SEM-EDS was conducted using a JEOL JCM-7000 with an acceleration voltage of 15 kV. Powder X-ray diffraction data was obtained by Panalytical Emperean (Malvern Panalytical Ltd) (λ = 1.54 Å), SmartLab (Rigaku Co., Ltd.) (λ = 1.54 Å), or synchrotron radiation at Beamline BL44B2 (λ = 0.8 Å) at Super Photon Ring-8 GeV (SPring-8) in Japan. To prepare the samples, Zn_3BHT was finely ground in an agate mortar and packed in 0.5 mm diameter soda tubes. PXRD simulations were obtained using the VESTA program.

XPS data were obtained using PHI 5000 VersaProbe (ULVAC-PHI, Inc.) with Al Kα (15 kV, 25 W) X-ray focused on a 100 μm^2 area. The spectra were analysed with MultiPak Software and standardized using the C(1s) peak at 284.6 eV. AFM measurements were carried out using an Agilent Technologies 5500 Scanning Probe Microscope, under ambient conditions, using the high-amplitude mode (tapping mode), with a silicon cantilever PPP-NCL (Nano-World). KFM measurements were carried out using an Agilent Technologies 5500 Scanning Probe Microscope, under Ar atmosphere, with a PtSi cantilever PtSi-FM (Nano-World). Conductivity measurements were performed using Au tips (purchased from ESSTech.) connected to a Keithley 2450 Sourcemeter. The measurement setup consisting of micromanipulators mounted on a measuring table placed on top of an anti-vibration stage and the entire setup enclosed in a glovebox, was custom ordered from Oyama Co. Ltd. Thermoelectric measurements to calculate Seebeck coefficient were performed using ZEM-

3. Raman spectroscopy was performed using a JASCO NRS-5500 Raman spectrometer with 532 nm laser irradiation.

II. Experimental Procedures

Liquid-liquid interfacial synthesis of Zn_3BHT for PXRD studies

In a glove box, 3.0 mg of BHT was added to degassed chloroform (10 mL) in a 50 mL glass vial. Degassed pure water (10 mL) was then layered over the chloroform solution and the mixture heated at 45 °C in a heat bath. $Zn(OAc)_2 \cdot 2H_2O$ (6.1 mg, 0.033 mmol) dissolved in 0.5 mL water was added dropwise and the mixture allowed to react at 45 °C overnight. A white film formed at the liquid-liquid interface, which was insoluble in any solvent. The water and chloroform layers were washed with pure degassed water and chloroform, respectively, followed by addition of degassed ethanol to homogenize the phases to quench the reaction. The nanosheets were filtered with a filter membrane and dried under vacuum.

For PXRD studies, the collected Zn_3BHT was added to metal ion solutions of $Cu(NO_3)_2 \cdot 3H_2O$ (50 mM) or $FeSO_4 \cdot 7H_2O$ (50 mM) and left to stand for 1 week. The resulting transmetallated product was filtered, washed with water and ethanol, and dried under vacuum before PXRD measurements.

Liquid-liquid interfacial synthesis of thin Zn_3BHT for heterojunction fabrication at 45°C

Under Ar atmosphere, a saturated solution of BHT in chloroform was prepared by adding 20 mL of degassed chloroform to 3.0 mg of BHT. The solution was shaken and the excess BHT filtered. A 2.5 cm x 1.3 cm SiO_2/Si substrate (or 2.0 x 2.0 cm Au/mica) was placed at an angle in a 20 mL vial. Degassed chloroform (5 mL) was added, followed by saturated BHT solution (5 mL). 5 mL of degassed water was layered over the organic phase and the reaction was heated at 45 °C for 30 min. Then Zn^{2+} solution (2.0 mM, 5 mL) was added carefully to the aqueous phase. After 10 minutes, the organic phase was removed to deposit the nanosheet on the substrate. The reaction was allowed to cool for 1 h. Degassed ethanol (5 mL) was then carefully layered on top of the aqueous phase. All the solvents were removed by a glass pipette, and the deposited thin Zn_3BHT film was washed with degassed chloroform. The film was annealed using a heat bath in the glovebox overnight at 70 °C. SEM-EDS, AFM, KFM, Raman, XPS results were obtained for samples synthesized based on this fabrication method.

Liquid-liquid interfacial synthesis of thin Zn_3BHT for heterojunction fabrication at RT

Under Ar atmosphere, a saturated solution of BHT in dichloromethane was prepared by adding 20 mL of degassed dichloromethane to 3.0 mg of BHT. The solution was shaken and the excess BHT filtered. A 2.5 cm x 1.3 cm SiO_2/Si substrate (or 2.0 x 2.0 cm Au/mica)

was placed at an angle in a 20 mL vial. Degassed dichloromethane (5 mL) was added, followed by saturated BHT solution (5 mL). 5 mL of degassed water was layered over the organic phase. Then Zn^{2+} solution (2.0 mM, 5 mL) was added carefully to the aqueous phase. After 10 minutes, the organic phase was removed to deposit the nanosheet on the substrate. Degassed ethanol (5 mL) was then carefully layered on top of the aqueous phase. All the solvents were removed by a glass pipette, and the deposited thin Zn_3BHT film was washed with degassed dichloromethane. The film was annealed using a heat bath in the glovebox overnight at 70 °C. Conductivity of tmCu, tmFe and diode measurements were carried out for samples synthesized based on this fabrication method.

Liquid-liquid interfacial synthesis of thin Zn_3BHT at 45°C for thermoelectric measurements

Under Ar atmosphere, glass substrates were placed in a 300-mL conical flask followed by degassed chloroform (50 mL) and BHT (1.6 mg in 2 mL chloroform) was carefully added. 50 mL of degassed water was layered over the organic phase and the reaction heated at 45 °C for 30 min. $Zn(OAc)_2 \cdot 2H_2O$ (3.7 mg in 1 mL H_2O) was added dropwise to the aqueous phase. After reacting overnight at 45 °C, the organic phase was removed to deposit the nanosheet on the substrate. The reaction was allowed to cool for 1 h. Degassed ethanol (50 mL) was then carefully layered on top of the aqueous phase. All the solvents were removed by a glass pipette, and the deposited thin Zn_3BHT film was washed with degassed chloroform. The film was annealed using a heat bath in the glovebox overnight at 70 °C. The samples were transmetallated respectively by immersing in $Cu(NO_3)_2 \cdot 7H_2O$ (50 mM) for 15 mins or $FeSO_4 \cdot 7H_2O$ (50 mM) solution for 7 days. The respective metal solutions were then carefully removed by pipette, the samples washed with 1:1 water:ethanol and left to dry in the glovebox overnight at RT.

Area-specific transmetallation of thin Zn_3BHT

Under an Ar atmosphere, a small substrate sample with deposited thin Zn_3BHT was placed in a sample tube, then either $Cu(NO_3)_2 \cdot 3H_2O$ or $FeSO_4 \cdot 7H_2O$ (50 mM) solution was added to partially immerse the substrate. The sample vial was then capped and left to stand for 3 days in the glove box. The metal solution was then carefully removed by pipette, the sample washed with 1:1 water:ethanol and left to dry in the glovebox overnight at RT.

Area-specific transmetallation of tmFe/Zn_3BHT to form tmFe/tmCu heterojunction

Under an Ar atmosphere, a small SiO_2/Si substrate sample with previously transmetallated tmFe/Zn_3BHT was placed in a sample tube, then aqueous $Cu(NO_3)_2 \cdot 3H_2O$ (50 mM) was carefully added up to the visible boundary between tmFe and ZnBHT. The

sample vial was then capped and left to stand for 3 days. The Cu(II) solution was then removed, the sample washed with 1:1 water:ethanol and left to dry in the glovebox.

III. Experimental Results

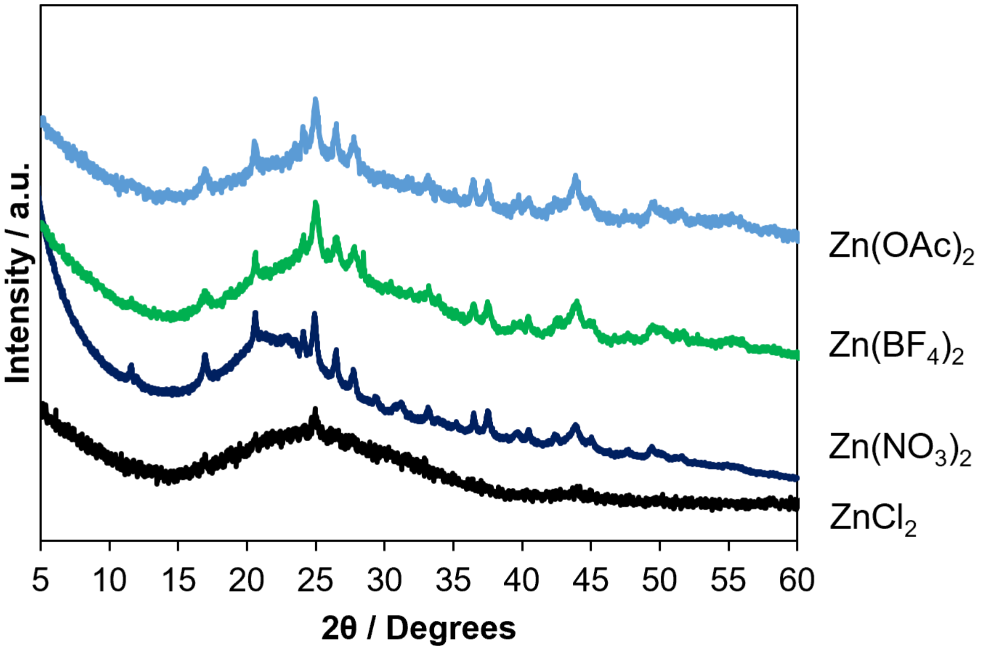

Figure S1. PXRD patterns of Zn_3BHT synthesized from various zinc salts (λ = 1.54 Å).

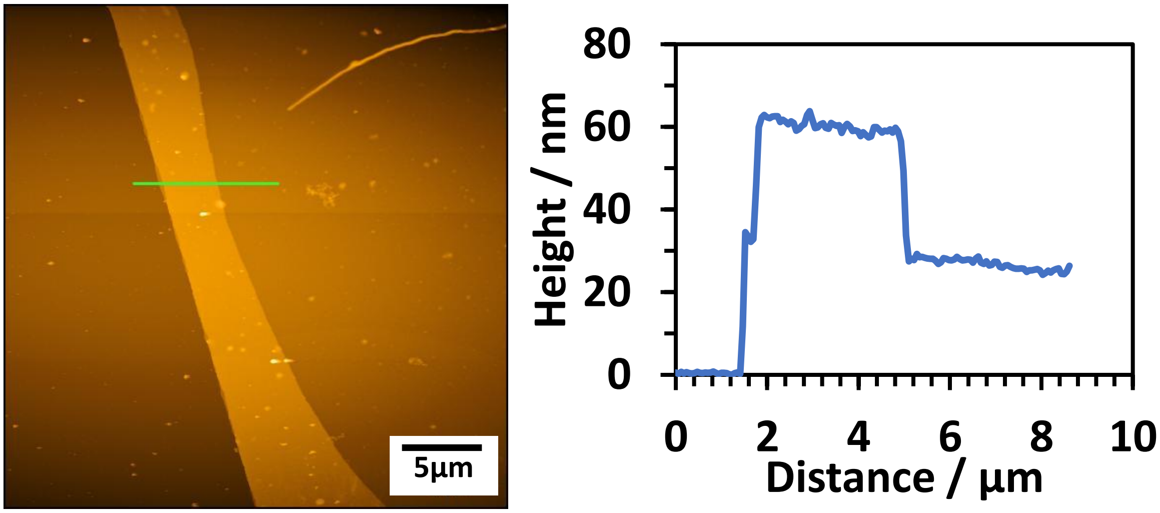

Figure S2. AFM topography image of Zn_3BHT and the height analysis at the green line.

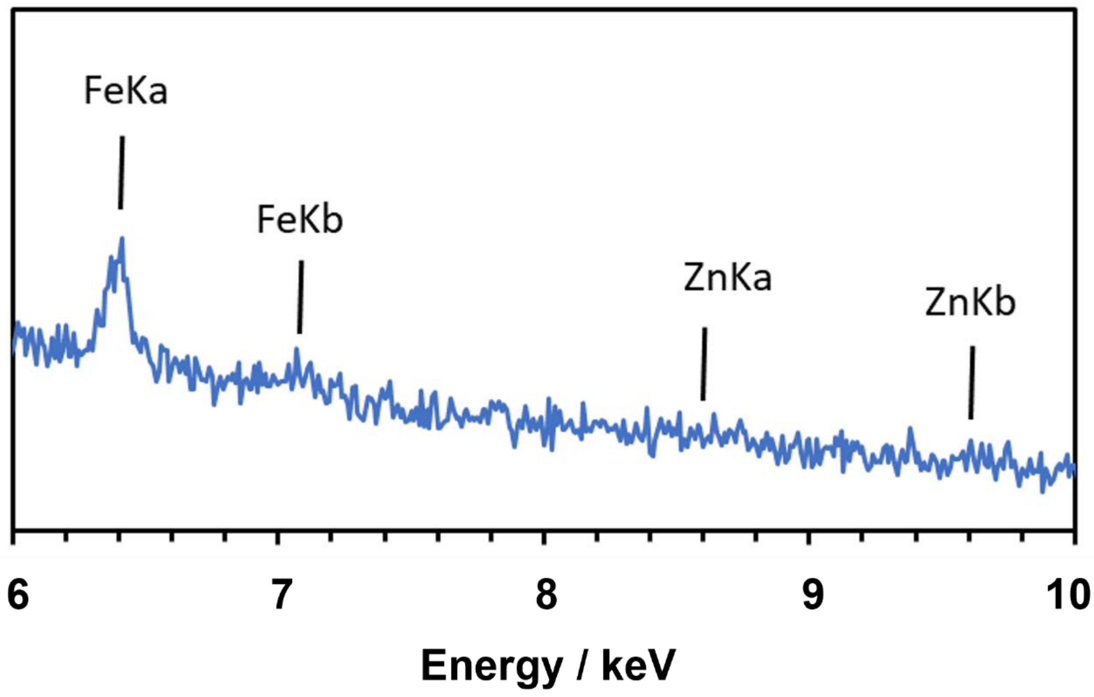

Element	Line	Mass%	Atom%
C	K	10.75±0.21	19.13±0.38
O	K	22.94±0.23	30.65±0.31
Si	K	65.22±0.21	49.63±0.16
S	K	0.64±0.03	0.43±0.02
Fe	K	0.42±0.04	0.16±0.02
Cu	K	0.02±0.03	0.01±0.01
Zn	K	Not detected	Not detected
Total		100.00	100.00

Figure S3. EDS energy spectrum (top) and atomic ratio (bottom) of tmFe.

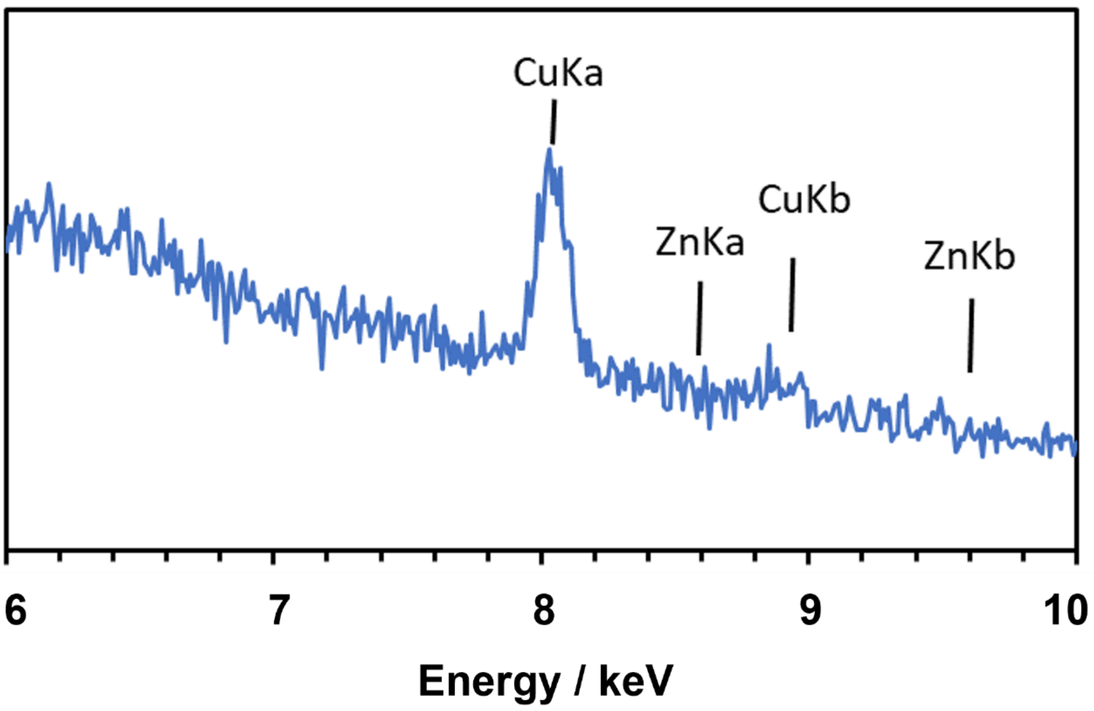

Element	Line	Mass%	Atom%
C	K	12.28±0.17	21.78±0.31
O	K	21.45±0.18	28.56±0.23
Si	K	64.14±0.16	48.64±0.12
S	K	0.93±0.03	0.62±0.02
Fe	K	0.03±0.01	0.01±0.01
Cu	K	1.16±0.08	0.39±0.03
Zn	K	Not detected	Not detected
Total		100.00	100.00

Figure S4. EDS energy spectrum (top) and atomic ratio (bottom) of tmCu.

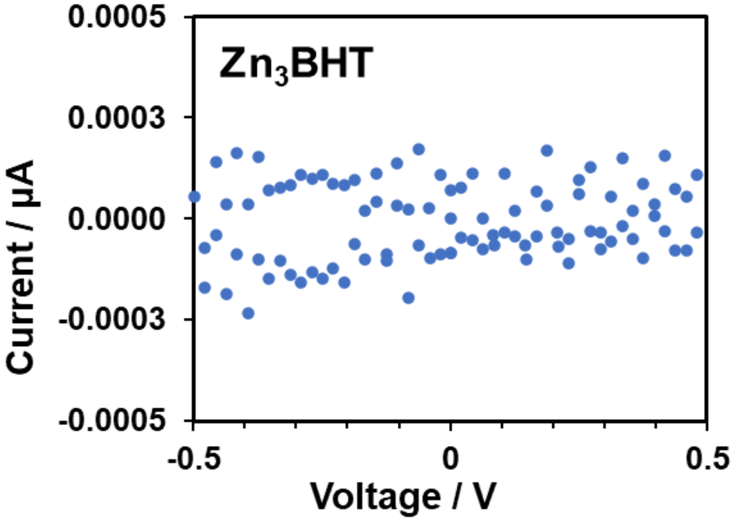

Figure S5. I-V curve of Zn_3BHT.

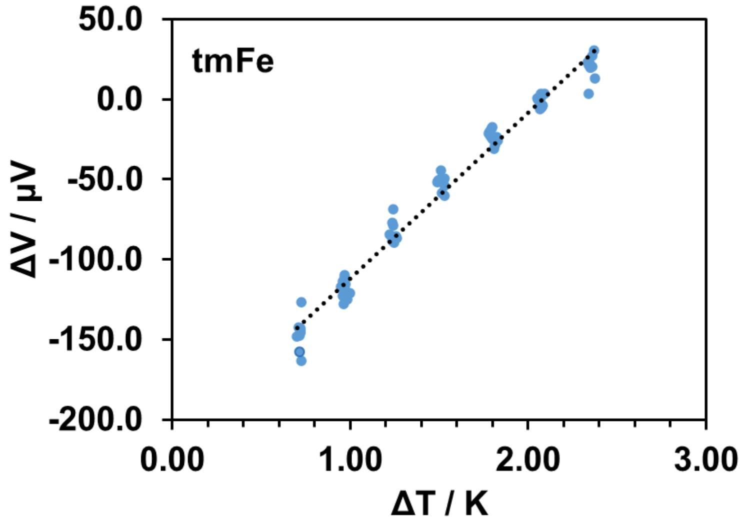

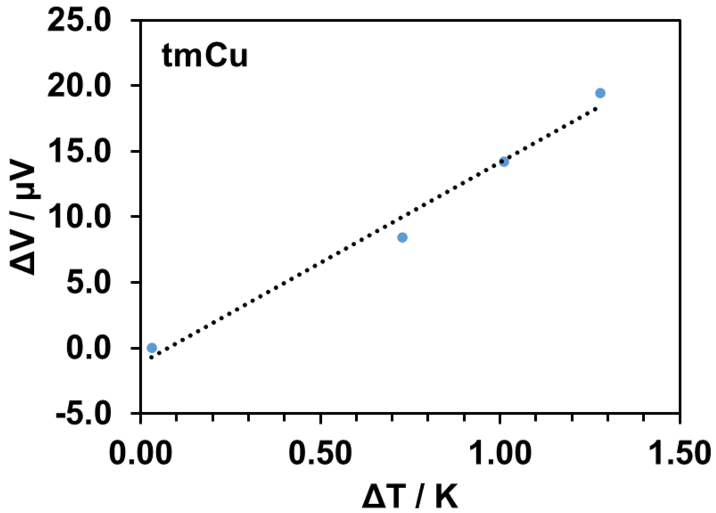

Figure S6. ΔV-ΔT plots of tmFe and tmCu.

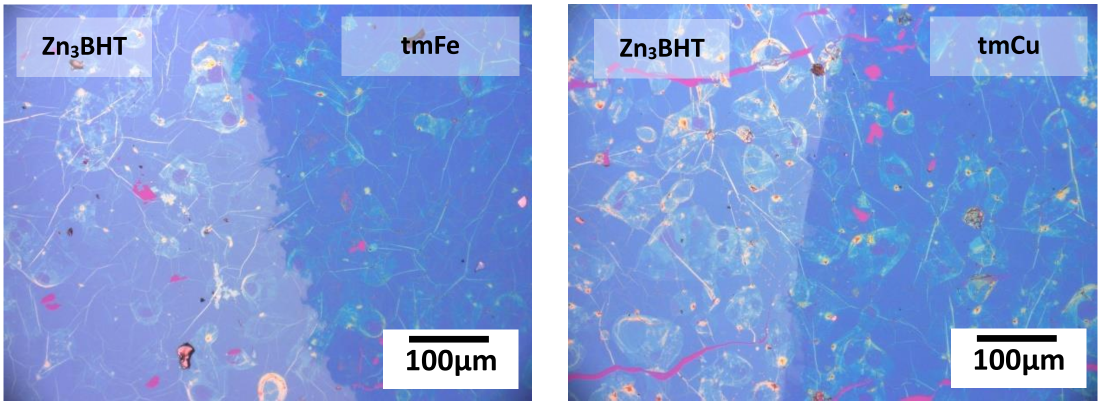

Figure S7. Optical images of Zn_3BHT/tmFe and Zn_3BHT/tmCu on silicon substrates.

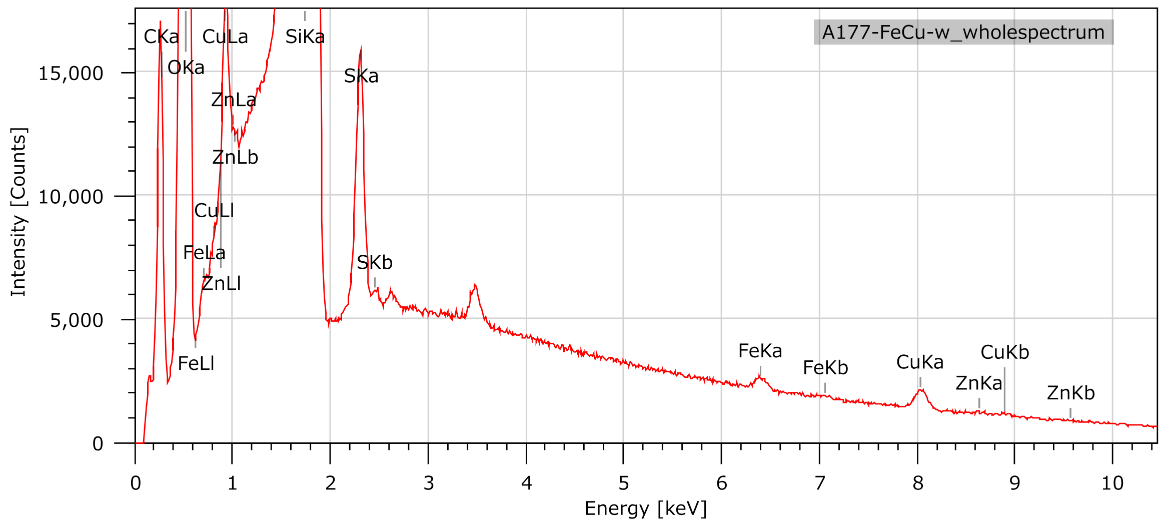

Figure S8. SEM-EDS mapping energy spectrum for tmFe/tmCu.

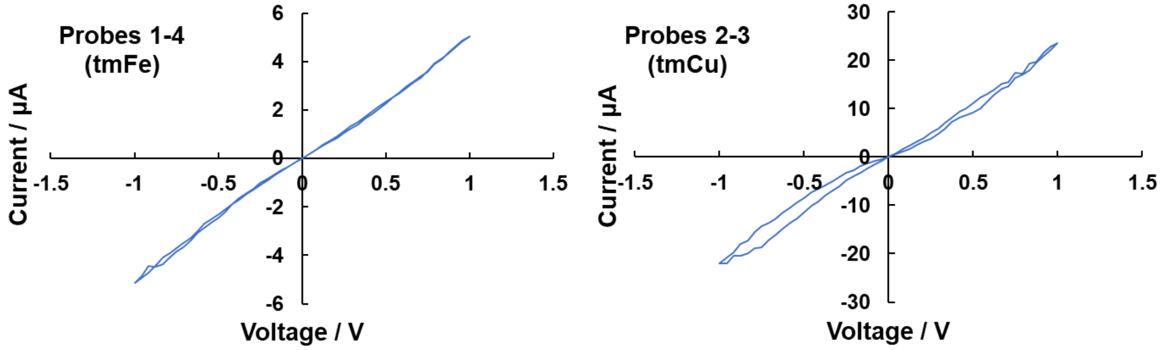

Figure S9. I-V curves measured between the 2 probes within each respective tmFe or tmCu region indicating ohmic behaviour.

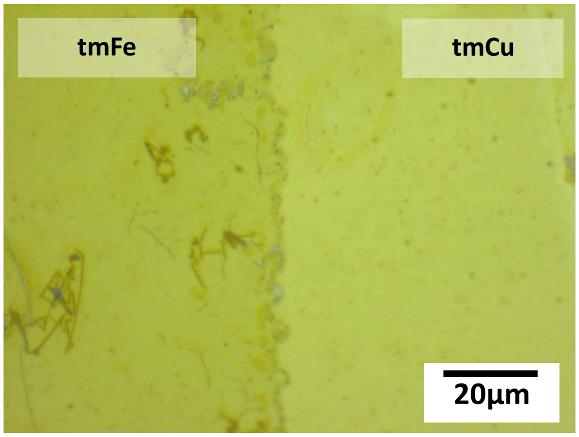

Figure S10. Optical image of tmFe/tmCu heterojunction on an Au/mica for KFM studies.

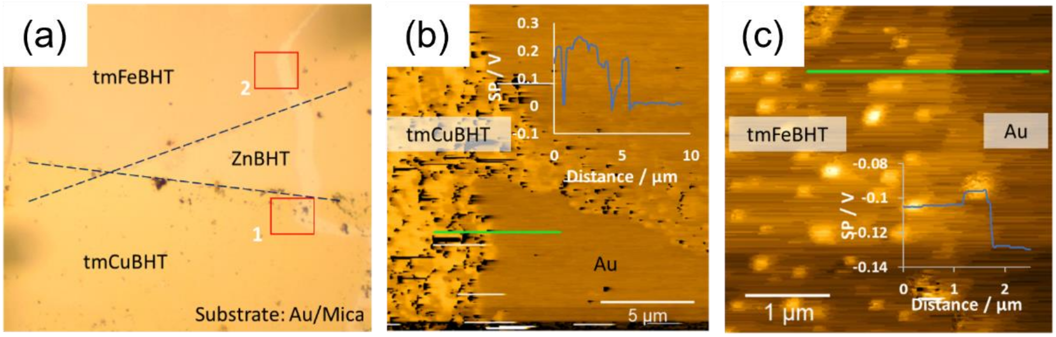

Figure S11. (a) An optical microscope image of tmFe/tmCu heterojunction on Au/mica. (b, c) KFM image measured (b) within the open red box 1 as shown in (a) for tmCu/Au junction and (c) within the open red box 2 for tmFe/Au junction.

IV. Reference

[S1] J. A. Harnisch, R. J. Angelici, *Inorganica Chim. Acta* **2000**, *300–302*, 273–279.
